# Integrating bioinformatic analysis and detailed experiments reveal an EMT‐related biomarker for clear cell renal cell carcinoma

**DOI:** 10.1002/cam4.6504

**Published:** 2023-09-07

**Authors:** Yue Ge, Sheng Ma, Junbiao Zhang, Zezhong Xiong, Beining Li, Siquan Ma, Bo Liu, Xiangyang Yao, Zhihua Wang

**Affiliations:** ^1^ Department of Urology, Tongji Hospital, Tongji Medical College Huazhong University of Science and Technology Wuhan China; ^2^ Department of Oncology, Tongji Hospital, Tongji Medical College Huazhong University of Science and Technology Wuhan China; ^3^ Department of Urology Zhongnan Hospital of Wuhan University Wuhan China

**Keywords:** biomarker, clear cell renal cell carcinoma, epithelial–mesenchymal transition, OLFML2B

## Abstract

**Background:**

Epithelial–mesenchymal transition (EMT) is associated with early recurrence and a poor prognosis in clear cell renal cell carcinoma (ccRCC). Studies have shown that EMT‐related genes play an important regulatory role in tumor invasion, metastasis, and drug resistance, but the biological functions of EMT‐related genes in ccRCC have not been specifically described.

**Methods:**

The mRNA and clinicopathological data of 532 ccRCC and 72 normal samples were downloaded from The Cancer Genome Atlas as a training set. The gene expression matrix and survival data of 91 and 101 ccRCC samples were obtained from the International Cancer Genome Consortium and the ArrayExpress databases as validation sets, respectively. Univariate Cox analysis was used to identify and cluster prognostic genes, and multivariate Cox was performed to construct a prognostic signature. Moreover, CIBERSORT and CellMiner were used to assess immune cell infiltration and prognostic gene‐drug sensitivity of the signature, respectively. Most importantly, we performed detailed experiments to verify the oncogenic function of a significant gene, OLFML2B, in vitro and in vivo.

**Results:**

We constructed a prognostic signature including seven genes and divided patients into high‐risk and low‐risk groups. The prognosis of the high‐risk group was significantly worse than that of the low‐risk group through Kaplan–Meier survival analysis. Interestingly, significant differences were observed in clinical characteristics and immune cell infiltration between the two groups. In addition, a significant correlation was found between the expression of prognostic genes and the sensitivity of tumor cells to chemotherapeutics. Most importantly, OLFML2B was proved to contribute to the proliferation and metastasis of ccRCC through detailed functional experiments in vitro and in vivo, and its prognostic efficacy for ccRCC patients was affirmed.

**Conclusion:**

We identified the prognostic signature of seven genes based on EMT‐related genes as prognostic biomarkers for ccRCC. Besides, OLFML2B was validated as a potential diagnostic and therapeutic target for ccRCC by our detailed experiments.

## 1 INTRODUCTION

Renal cell carcinoma (RCC) is a type of cancerous tumor originating from the renal epithelium, which accounts for 2.2% of adult malignant tumors.^1^ The incidence of RCC varies between countries and regions, with a higher incidence in developed countries (accounting for 4.2% of newly diagnosed cancers).^2^ According to recent research, approximately 73,750 cases were diagnosed with renal cancer and renal pelvis cancer, and 14,830 cases would eventually die in the United States in 2020.^3^ Clear cell renal cell carcinoma (ccRCC) is the most common lethal subtype of RCC, accounting for 70%–80% of all RCC cases.^4^, ^5^ For patients with early or local ccRCC, radical surgical resection remains the first choice in clinical treatment, but surgery is not suitable for advanced or metastatic tumors.^6^ In addition, 30% of patients had late metastases after receiving early surgery.^7^ Furthermore, ccRCC is insensitive to radiotherapy and chemotherapy. Late complications and adverse reactions caused by radiotherapy and chemotherapy have always plagued patients and clinicians.^8^ With the extensive research on the molecular biology of ccRCC, targeted therapy programs represented by immunotherapy have been widely accepted by clinicians and patients, and have already achieved certain curative effects.^9^ However, targeted therapy resistance may lead to poor survival results in considerable proportion of ccRCC patients.^10^ The identification and investigation of novel biomarkers is imperative for the advancement of clinical approaches in diagnosing, prognosticating, and targeting therapeutic interventions toward ccRCC. Hence, the exploration of potential unexplored biomarkers holds substantial significance.

Epithelial–mesenchymal transition (EMT), the process of phenotypic transformation from epithelial cells to mesenchymal cells, is an essential step in embryonic development and cancer metastasis.^11^, ^12^ EMT involves loss of polarity and impaired adhesion abilities in epithelial cells, while the expression of mesenchymal cell markers enables them to gain the ability of invasion and migration.^13^ Studies on the involvement of EMT in the regulation of tumor metastasis and therapeutic resistance have been continuously reported, including ccRCC.^14^, ^15^, ^16^ In addition, EMT has an important regulatory effect on tumor cell drug resistance.^17^ Cells that have undergone EMT show similar characteristics to cancer stem cells, possessing the same signaling pathways and drug‐resistant phenotypes as cancer stem cells.^18^ Interestingly, studies have shown that the characteristics of cancer mesenchymal transformation, and the simultaneous analysis of the adjacent microenvironment, have clarified the potential mechanism of cancer invasion.^19^, ^20^ Therefore, the study of EMT‐related genes is likely to be a potential therapeutic target in the future. However, current researches on the prognostic evaluation, diagnosis, and treatment of ccRCC by EMT‐related genes have not been studied, and it is worthy of further exploration.

In this study, we aimed to delineate a prognostic model for ccRCC utilizing expression analysis of EMT‐related genes in samples obtained from The Cancer Genome Atlas (TCGA). Cluster analysis was performed on the gene expression data, and subsequent validation was carried out using the external data sets International Cancer Genome Consortium (ICGC) and ArrayExpress. In addition, further investigations of these EMT‐associated genes were conducted to infer their potential roles in immune infiltration and drug resistance. Finally, the *OLFML2B* gene, which was of great significance to our analysis and rarely studied, was selected for functional validation in vitro and in vivo. These results may provide new perspectives for the diagnosis and development of therapeutic targets for ccRCC.

## 2 MATERIALS AND METHODS

### 2.1 Data acquisition

The training set (KIRC) (https://xenabrowser.net) encompassing 532 ccRCC specimens and 72 normal samples was procured from the TCGA database to obtain transcriptome data for RNA‐seq in conjunction with the corresponding clinical characteristics. Gene expression matrix and survival information for 91 ccRCC samples were collected from the ICGC database (RECA‐EU/FR) (https://dcc.icgc.org/projects/RECA‐EU). Additionally, 101 ccRCC patients from the ArrayExpress database (E‐MTAB‐1980 dataset) were included in the downstream analysis. Furthermore, 99 EMT‐related genes were downloaded from the MSigDB database (http://www.broad.mit.edu/gsea/msigdb/) (Table S1).

### 2.2 Tumor microenvironment evaluation

For analyzing immune infiltration, CIBERSORT is one of the most commonly used tools, which can provide the percentage of single cells in a mixed cell population.^21^ We sourced a list of 22 immune cells as the gene signature matrix, and their respective proportions were evaluated in both the high‐risk and low‐risk groups by concomitant analysis with the gene expression matrix.

### 2.3 Sensitivity analysis of chemotherapy

The NCI‐60 cell line is the standard model for preclinical evaluation of anticancer drugs owing to its extensive utilization. RNA sequencing data for 60 distinct cancer cell lines and their corresponding drug activity profiles were sourced from the CellMiner database.^22^ Subsequently, Pearson correlation analysis was performed on the prognostic gene expression and sensitivity of 792 drugs approved by the FDA or in clinical trials (Table S2).

### 2.4 Cell lines and cell culture

The Shanghai Cell Bank Type Culture Collection Committee (Shanghai, China) provided the human ccRCC cell lines (786‐O and OS‐RC‐2) as well as the HEK‐293T cells. All cell culture methods and conditions were specifically introduced in our previous article.^23^

### 2.5 Construction of plasmids and transfections

Relative target sequences were inserted into the lentiviral vector pCDH‐MSCV‐MCS‐EF1‐copGFP (System biosciences, USA) and pLKO.1 plasmid (Addgene). All plasmids were confirmed by DNA sequencing (Tsingke Biotechnology). Lentivirus was produced in 293T cells by transfecting the above plasmids together with pHelper1.0, pHelper2.0, pHelper3.0 via Lipofectamine 3000 (Invitrogen). After 48 h of incubation, the lentivirus‐containing supernatant was filtered through 0.22 mm filters and transfected into target ccRCC cell lines. Finally, after antibiotic selection (puromycin, 2 mg/mL), surviving cells were used for further experiments.

### 2.6 Xenograft experiments

We purchased male BALB/c nude mice from Vital River Laboratory Animal Technology at the age of 4–5 weeks. For the subcutaneous injection experiments, 3.0 × 10^6^ 786‐O cells were suspended with PBS solution and injected subcutaneously into four nude mice per group at the right flank. A 7‐day measurement and calculation of tumor sizes were performed. After 4 weeks, mice were executed, and tumors were excised and weighed. Ethics review and approval were obtained from the Ethics Committee of Tongji Medical College, Huazhong University.

## 3 RESULTS

### 3.1 Identification of EMT‐related genes in ccRCC

**FIGURE 1:**
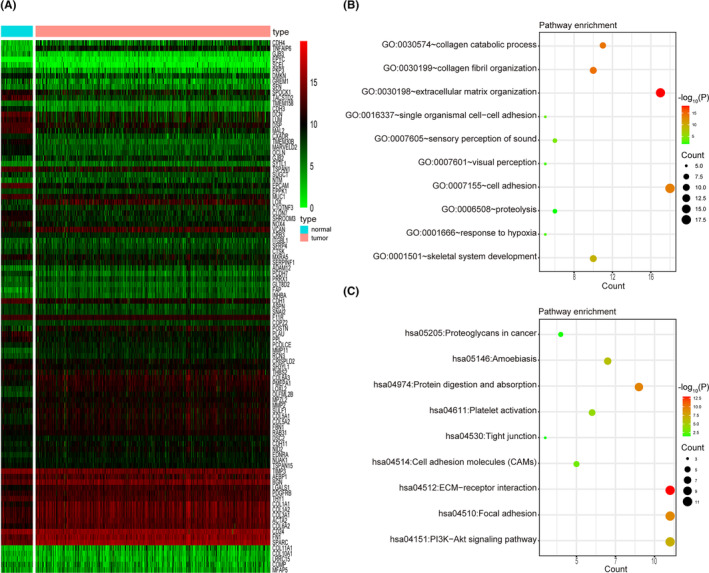
Prognostic‐related genes. (A) Heatmap showing the expression of 57 prognostic‐related genes between clear cell renal cell carcinoma and normal samples. (B) GO and (C) KEGG analysis of identified genes.

A total of 99 EMT‐related genes were extracted from the MSigDB database and subsequently aligned against an mRNA expression matrix comprising of 532 ccRCC samples sourced from the TCGA database. Univariate Cox analysis was used to identify 57 prognostic genes (FDR < 0.05) (Table S3), and their detailed expressions were shown in Figure 1A. GO and KEGG analysis showed that these genes were primarily enriched in extracellular matrix organization, ECM‐receptor interaction, and cell adhesion (Figure 1B,C).

### 3.2 Consistent clustering of ccRCC by EMT‐related genes

**FIGURE 2:**
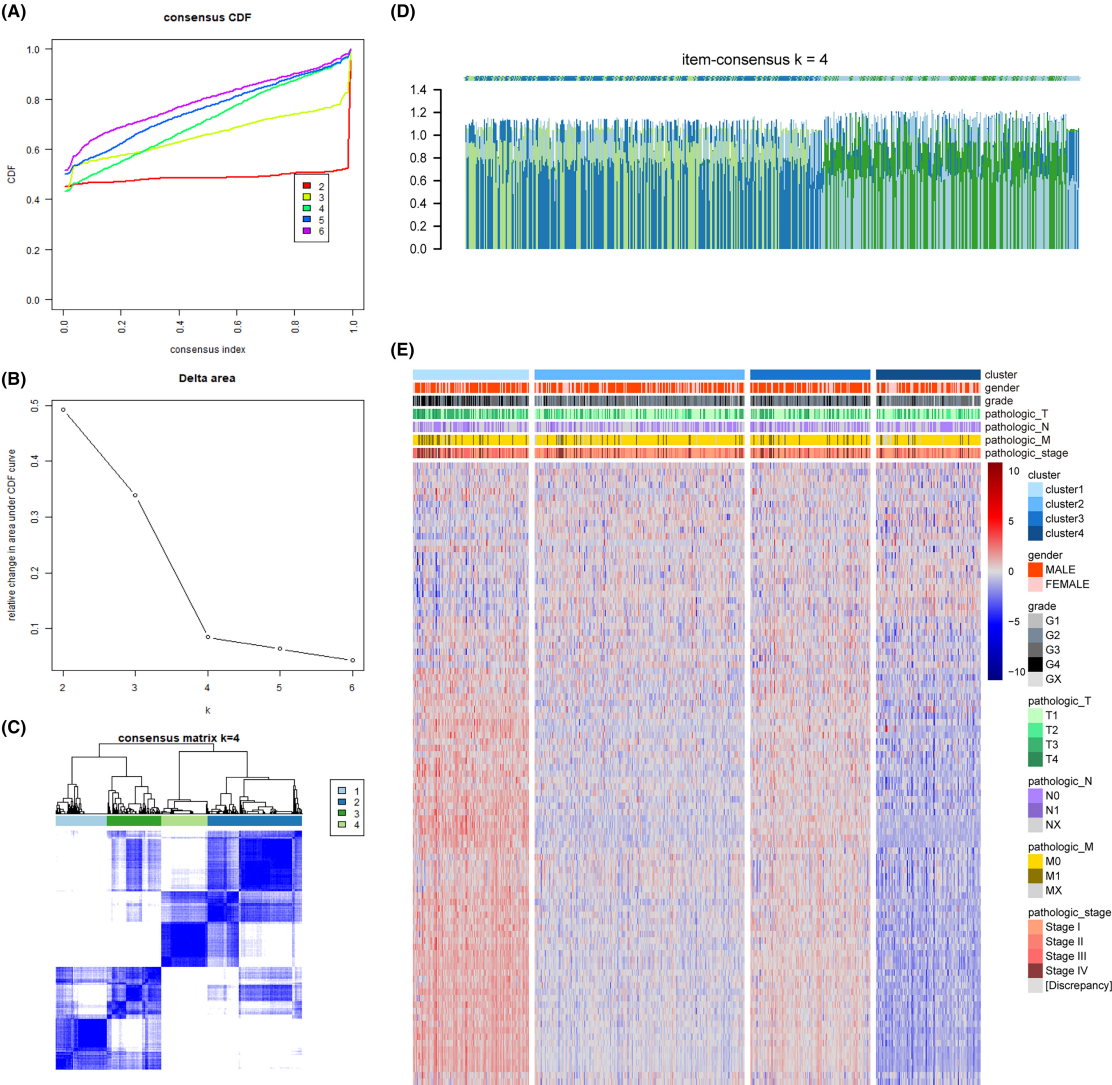
Consistent clustering of clear cell renal cell carcinoma (ccRCC) based on prognostic‐related genes. (A) Cumulative distribution function (CDF) curve. (B) Delta area curve, showing the change of area under the CDF curve between *K* and *K* − 1. (C) The matrix heatmap for *K* = 4. The value of consistency matrix from 0 to 1 represents the color from white to blue. (D) Item‐Consensus Plot for *K* = 4. Each bar graph represents a sample, to demonstrate the purity of the samples. (E) Heat map that categorizes ccRCC patients into four clusters based on prognostic‐related genes.

An unsupervised clustering analysis was performed to explore the predictive impact of 57 prognostic genes on the clinical characteristics and prognosis of patients with ccRCC. Utilizing the R package ConsensusClusterPlus, the 57 EMT‐related genes were consistently clustered, and the optimal number of clusters was determined through the implementation of cumulative distribution function (CDF). Combining CDF and delta area facilitated the identification of *K* = 4 as the number of clusters providing the highest consistency and clustering confidence since it corresponds to a stable descending slope in the CDF curve (Figure 2A,B). The item‐consensus graph demonstrated that the classification region of the samples remained consistent and sufficiently homogeneous when the number of clusters was designated as four (Figure 2D). The matrix heatmap offered a visual representation that portrayed the composition of samples in the cluster (Figure 2C). Finally, the heatmap of 57 EMT‐related genes in four clusters was shown in Figure 2E.

**FIGURE 3:**
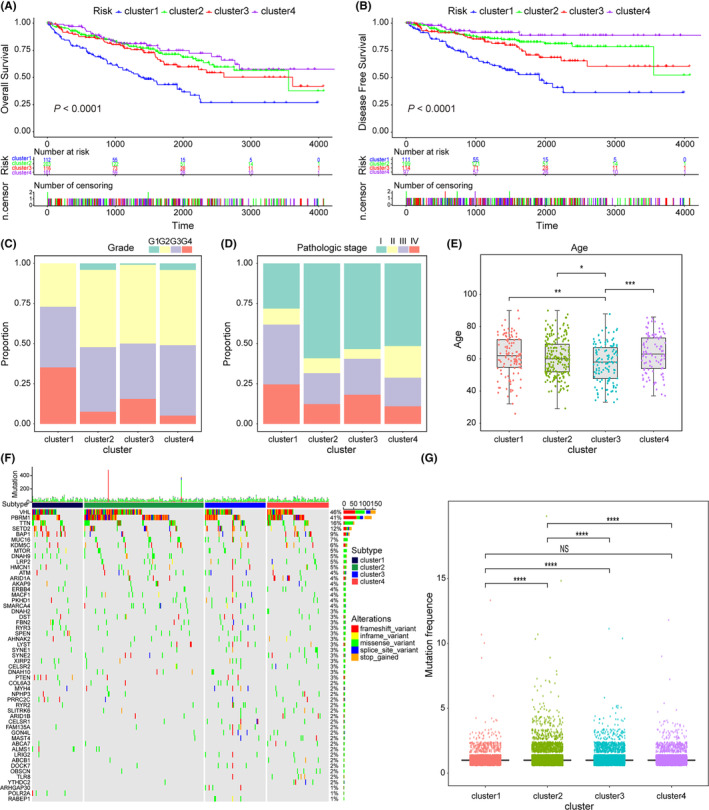
Characterization of different features of prognostic‐related genes clustering. (A, B) Kaplan–Meier survival curves showed the difference of the overall survival (OS) and disease‐free survival (DFS) between the four clusters. (C–E) Proportion of clinical of features in four clusters. (F, G) The differences of somatic mutations among four clusters. NS, not significant, *p < 0.05; **p < 0.01; ***p < 0.001; ****p < 0.0001.

Furthermore, to evaluate the overall survival (OS) and disease‐free survival (DFS) of the four clusters, a Kaplan–Meier curve analysis was conducted and indicated that, in comparison to the other clusters, cluster 1 demonstrated the poorest prognosis, while cluster 4 exhibited the most favorable prognosis (Figure 3A,B), which is consistent with the results we observed in the validation set (Figure S1). Subsequently, we further analyzed the clinical characteristics of the four clusters, including grade, stage, and age. Cluster 1 and cluster 3 had a higher degree of grade and stage, while cluster 2 and cluster 4 were relatively low, which was consistent with their prognosis (Figure 3C,D). It is worth noting that the age of cluster 3 was the lowest among the three groups, concentrated between 50 and 65 years old (Figure 3E). The waterfall plot indicated that cluster 2 presented a significantly higher mutation burden (Figure 3F). At the same time, the box plot showed that significant differences existed in the mutation frequency between the groups (Figure 3G).

### 3.3 Construction and validation of EMT‐related genes prognosis signature

**FIGURE 4:**
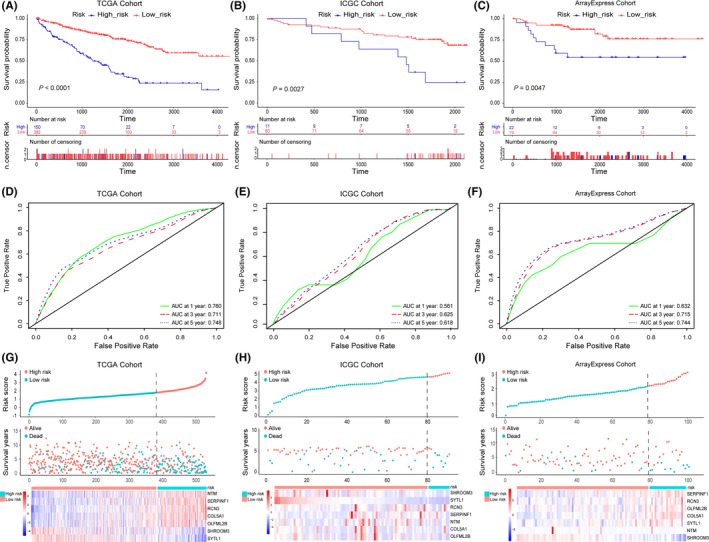
Construction and validation of prognostic signature. (A–C) The Kaplan–Meier survival curves of the prognostic signature between high‐ and low‐risk groups. (D–F) The time‐dependent ROC for 1‐, 3‐, and 5‐years for the overall survival in the training and validation datasets. (G–I) The distribution of risk score, survival status, and gene expression panel in the training and validation datasets.

To identify genes that were strongly related to OS, we further performed a multivariate Cox analysis on 57 prognostic‐related genes. Finally, seven prognostic‐related genes (*NTM*, *SERPINF1*, *RCN3*, *COL5A1*, *OLFML2B*, *SHROOM3*, and *SYTL1*) were identified to construct a prognostic signature (Table 1). Subsequently, a prognostic model was constructed: risk score = (0.14451639 × COL5A1 expression) + (0.13557763 × RCN3 expression) + (0.09432297 × NTM expression) + (0.18426511 × SERPINF1 expression) + (0.08420989 × SYTL1 expression) − (0.22129188 × OLFML2B) − (0.23281388 × SHROOM3 expression). Using the survminer package of R software, the optimal cut‐off value of the risk score was calculated to be 1.835, and then the ccRCC patients from TCGA were divided into high‐risk and low‐risk groups. The Kaplan–Meier survival curve revealed that the high‐risk group was associated with the poor OS when compared with the low‐risk group (*p* < 0.0001, Figure 4A). Additionally, the calculated area under the time‐dependent ROC curve for 1‐, 3‐, and 5‐year OS rates were determined to be 0.76, 0.711, and 0.748, respectively. These values indicate that the model exhibits favorable predictive capabilities (Figure 4D).

**TABLE 1:** Multivariate cox regression analysis for the seven transcription factors expression levels in in TCGA KIRC cohort.

Genes	HR (95% CI)	*p* value
OLFML2B	1.666 (1.500–2.413)	0.006
COL5A1	3.042 (1.847–5.008)	<0.001
SHROOM3	0.759 (0.649–0.886)	<0.001
SERPINF1	1.387 (1.103–1.743)	0.005
NTM	1.187 (1.048–1.343)	0.007
RCN3	1.537 (1.117–2.112)	0.008
SYTL1	0.794 (0.669–0.943)	0.009

Abbreviations: CI, confidence interval; HR, hazard ratio; KIRC, kidney clear cell carcinoma; TCGA, The Cancer Genome Atlas.

**FIGURE 5:**
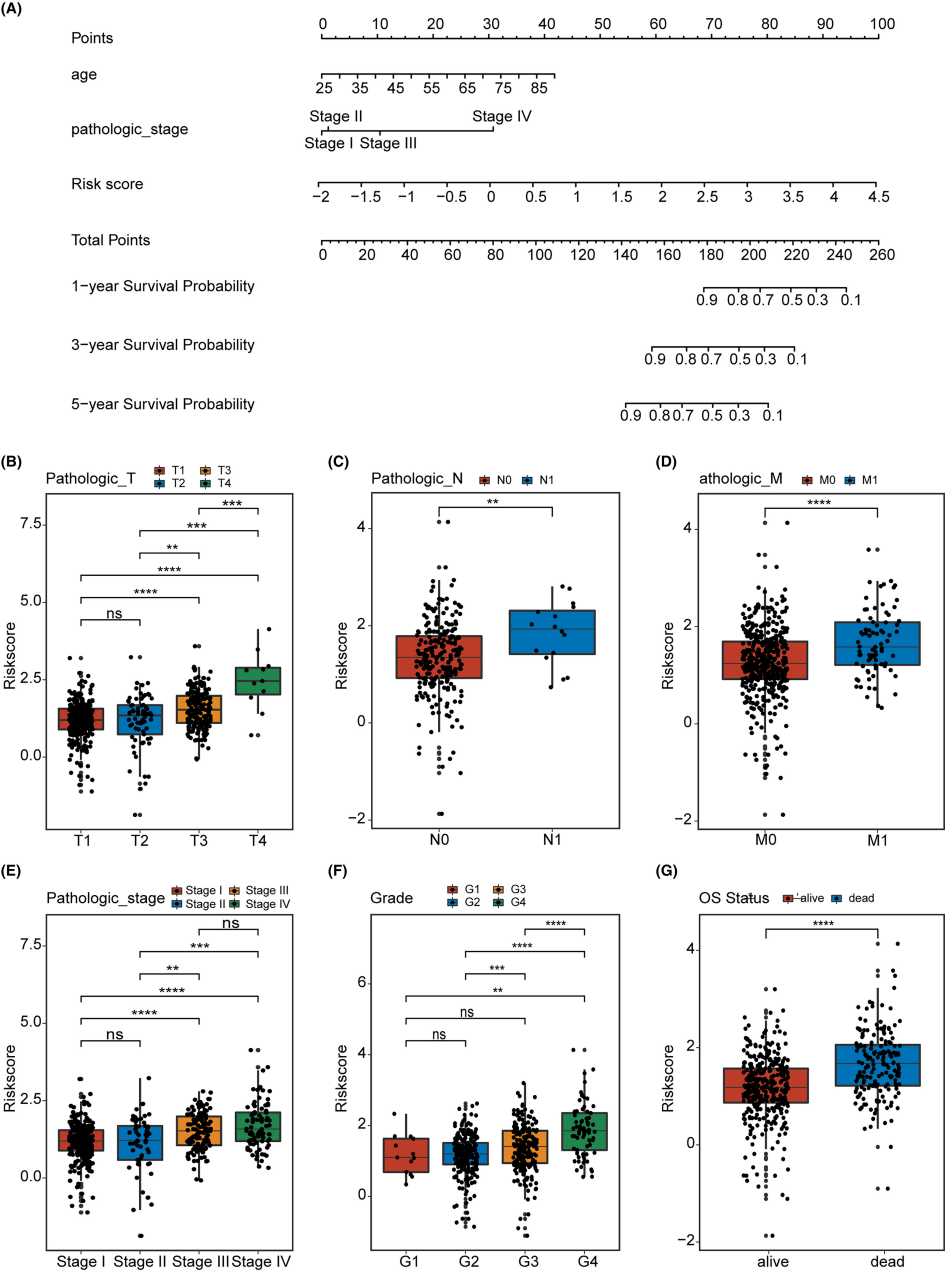
Construction of the nomogram and clinical relevance of prognostic signature. (A) Nomogram construction for the 1‐, 3‐, and 5‐ year overall survival (OS) prediction for the clear cell renal cell carcinoma. Association between the risk score of prognostic signature and clinical factors, including (B) T; (C) N; (D) M; (E) stage; (F) grade; (G) OS status. NS, not significant, *p < 0.05; **p < 0.01; ***p < 0.001; ****p < 0.0001.

To further assess the efficacy of the prognostic signature, we analyzed 91 samples of ccRCC from the ICGC database and 101 ccRCC patients from the ArrayExpress database. Using a consistent computational formula, we determined the risk score for each sample and categorized them into high‐risk or low‐risk groups accordingly. The OS of patients in the high‐risk group was significantly lower than that of the low‐risk group (Figure 4B,C). The AUCs of the seven‐gene signature for the 1‐, 3‐, 5‐year OS were 0.561, 0.625, and 0.618 in the ICGC database, and were 0.632, 0.715, and 0.744 in the ArrayExpress database (Figure 4E,F). Finally, the risk score, survival status, and gene expression profile of TCGA, ICGC, and ArrayExpress samples were shown in Figure 4G–I. Subsequently, we have established a predictive nomogram for OS at 1, 3, and 5 years to better predict the survival probability of individual (Figure 5A). Furthermore, to explore the relationship between EMT‐related genes signature and the clinical characteristics of ccRCC patients, the risk scores of TNM stage, pathological stage, tumor grade, and OS status were compared. Results suggested that higher T classification, more lymphatic metastasis, and distant metastasis were significantly associated with high‐risk scores (Figure 5B–D). At the same time, for patients with a higher tumor stage and grade, the risk score was higher (Figure 5E,F), and patients with high‐risk scores had poorer survival status compared patients with low‐risk scores (Figure 5G).

### 3.4 Immune infiltration in high‐risk and low‐risk group

**FIGURE 6:**
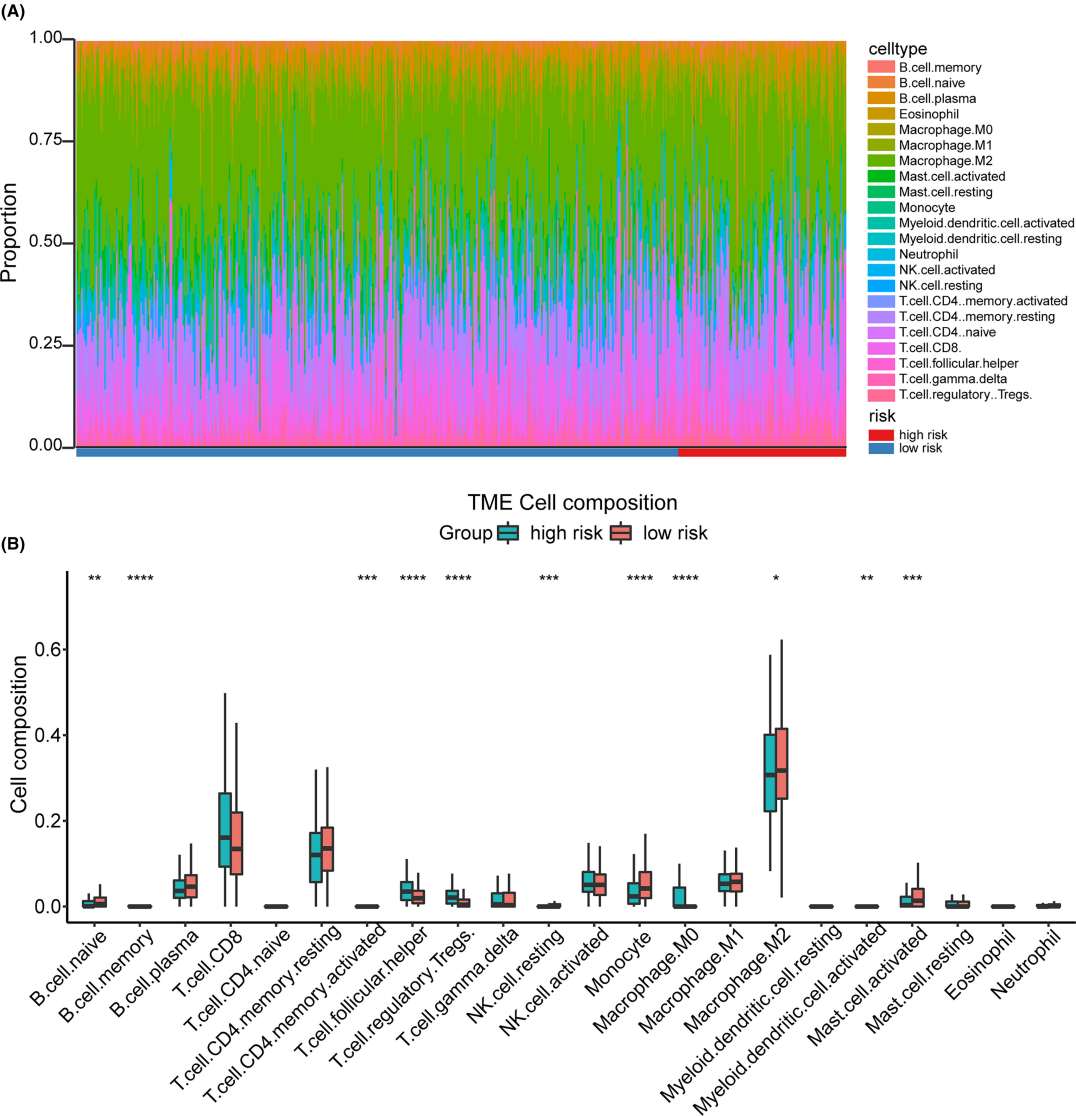
Immune landscape between high‐risk and low‐risk patients. (A) Proportion of immune cell infiltration in high‐ and low‐risk groups. (B) The percentage of different immune cells in the high‐ and low‐risk groups. *p < 0.05; **p < 0.01; ***p < 0.001; ****p < 0.0001.

The role of immune infiltration in tumor development and its impact on the clinical prognosis of cancer patients have been extensively reported in the tumor microenvironment (TME) literature.^24^ Research by Noman et al. showed that the expression of the immune checkpoint ligand PD‐L1 depends on the activation of EMT‐related genes in breast cancer.^25^ In RCC, tumor immune infiltration was closely related to clinical prognosis.^26^ Therefore, we investigated the ability of EMT‐related genes signature to assess the immune microenvironment. The CIBERSORT method was utilized to evaluate the immune infiltration status of 22 distinct immune cell types in high‐risk and low‐risk ccRCC samples (Figure 6A; Table S4). The results indicated that the high‐risk group presented significantly higher proportions of M0 Macrophage, activated memory CD4^+^ T cells, and Tregs, while B cells, Monocyte, and NK cells had the opposite behaviors (Figure 6B). These findings indicate that EMT‐related genes may play regulatory roles in immune cell infiltration within the tumor microenvironment of ccRCC.

### 3.5 Correlation between prognostic gene expression and chemotherapy sensitivity

**FIGURE 7:**
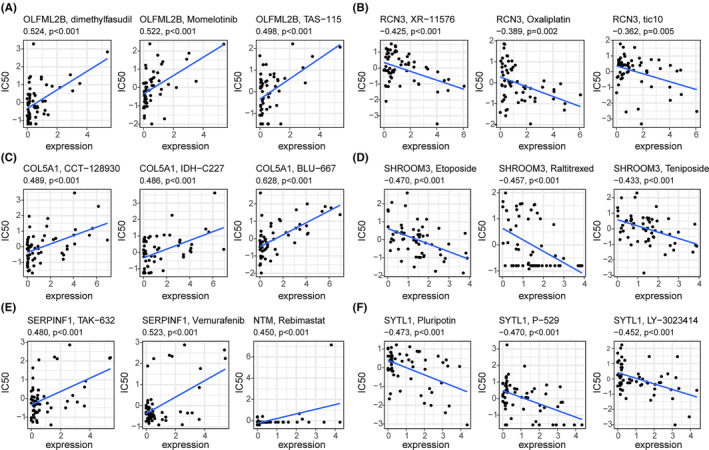
Scatter plot showing the relationship between prognostic gene expression and drug sensitivity. (A) OLFML2B. (B) COL5A1. (C) SERPINF1 and NTM. (D) RCN3. (E) SHROOM3. (F) SYTL1.

The clinical management of ccRCC is significantly impeded by its inherent resistance to chemotherapy. This obstacle may be attributed to heightened expression levels of prognostic genes within cancer cells, which exacerbate their resistance to chemotherapeutic agents. To explore this phenomenon further, we utilized the CellMiner database to obtain expression data pertaining to prognostic genes in the NCI‐60 cell line. Our subsequent analyses revealed a compelling correlation between the expression levels of all prognostic genes and chemotherapeutic sensitivities (*p* < 0.01, Figure 7). The increased expression of OLFML2B, COL5A1, SERPINF1, and NTM was related to the increased sensitivities of various chemotherapy drugs such as dimethylfasudil, momelotinib, TAS‐115, CCT‐128930, IDH‐C227, BLU‐667, TAK‐632, vemurafenib, and rebimastat (Figure 7A–C). In contrast, the high expression of RCN3, SHROOM3, and SYTL1 was associated with the increased resistance of drugs such as XR‐11576, oxaliplatin, tic10, etoposide, raltitrexed, afatinib, pluripotin, P‐529, and LY−3023414 (Figure 7D–F). These results indicated that certain prognostic genes may serve as viable therapeutic targets to address drug resistance or enhance adjuvant drug efficacy.

### 3.6 OLFML2B independently predicts the prognosis of ccRCC and may be involved in EMT process

**FIGURE 8:**
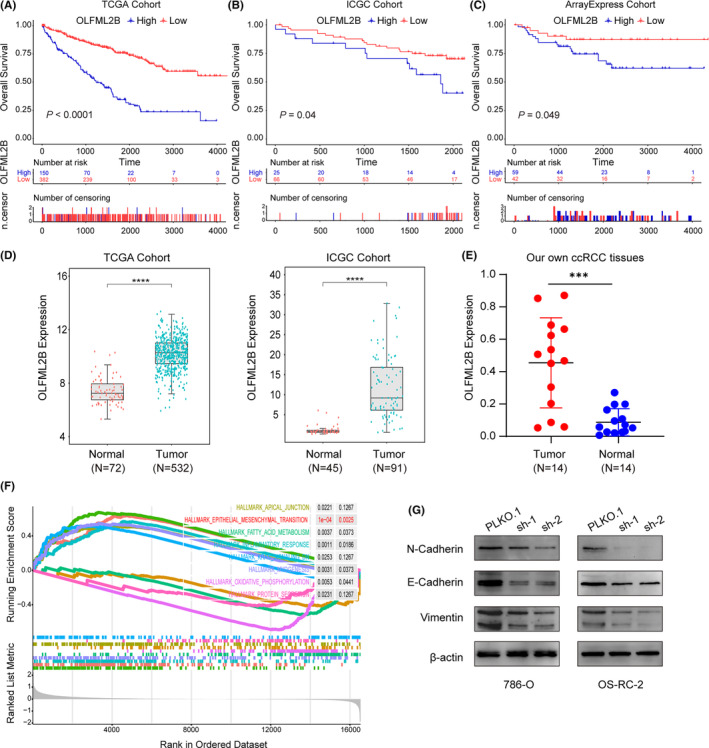
OLFML2B predicted poor prognosis in clear cell renal cell carcinoma (ccRCC) may through epithelial–mesenchymal transition (EMT). (A–C) Kaplan–Meier survival curves demonstrated that patients with high OLFML2B expression were associated with the worse overall survival (OS). (D, E) In The Cancer Genome Atlas database, International Cancer Genome Consortium database, and our own ccRCC tissues, OLFML2B was significantly overexpressed in tumor samples compared with normal samples. (F) The gene set enrichment analysis (GSEA) of hallmark gene sets in high‐expression‐group of OLFML2B. (G) OLFML2B promoted the expression of EMT pathway related proteins.

The prognostic regulation of COL5A1 on ccRCC has been confirmed in many studies^27^, ^28^ but OLFML2B, another important gene in the model, has not been explored yet. Kaplan–Meier curve showed that in both TCGA, ICGC, and ArrayExpress cohorts, patients exhibiting high OLFML2B expression levels displayed a significantly reduced OS compared to those with low expression (Figure 8A–C). Besides, our analysis of TCGA and ICGC cohorts revealed a significant upregulation of OLFML2B expression in tumor samples as compared to normal samples (Figure 8D). Consistently, we performed RT‐qPCR for OLFML2B in 14 paired tissues, and the result demonstrated that OLFML2B was highly expressed in tumor tissues (Figure 8E). Furthermore, to explore the underlying mechanism of OLFML2B, gene set enrichment analysis (GSEA) analysis was performed to evaluate biological processes and pathway enrichment of OLFML2B. The result indicated that increased expression of OLFML2B may lead to the activation of the EMT pathway (*p* < 0.001, Figure 8F). To verify the above analysis, ccRCC cell lines stably silencing OLFML2B were constructed, and their efficiency was validated (Figure S2). Convincingly, Western blot analysis was carried out to confirm the relationship between OLFML2B and the EMT pathway, and the results validated the initial hypothesis (Figure 8G).

### 3.7 OLFML2B promotes ccRCC progression in vitro and in vivo

**FIGURE 9:**
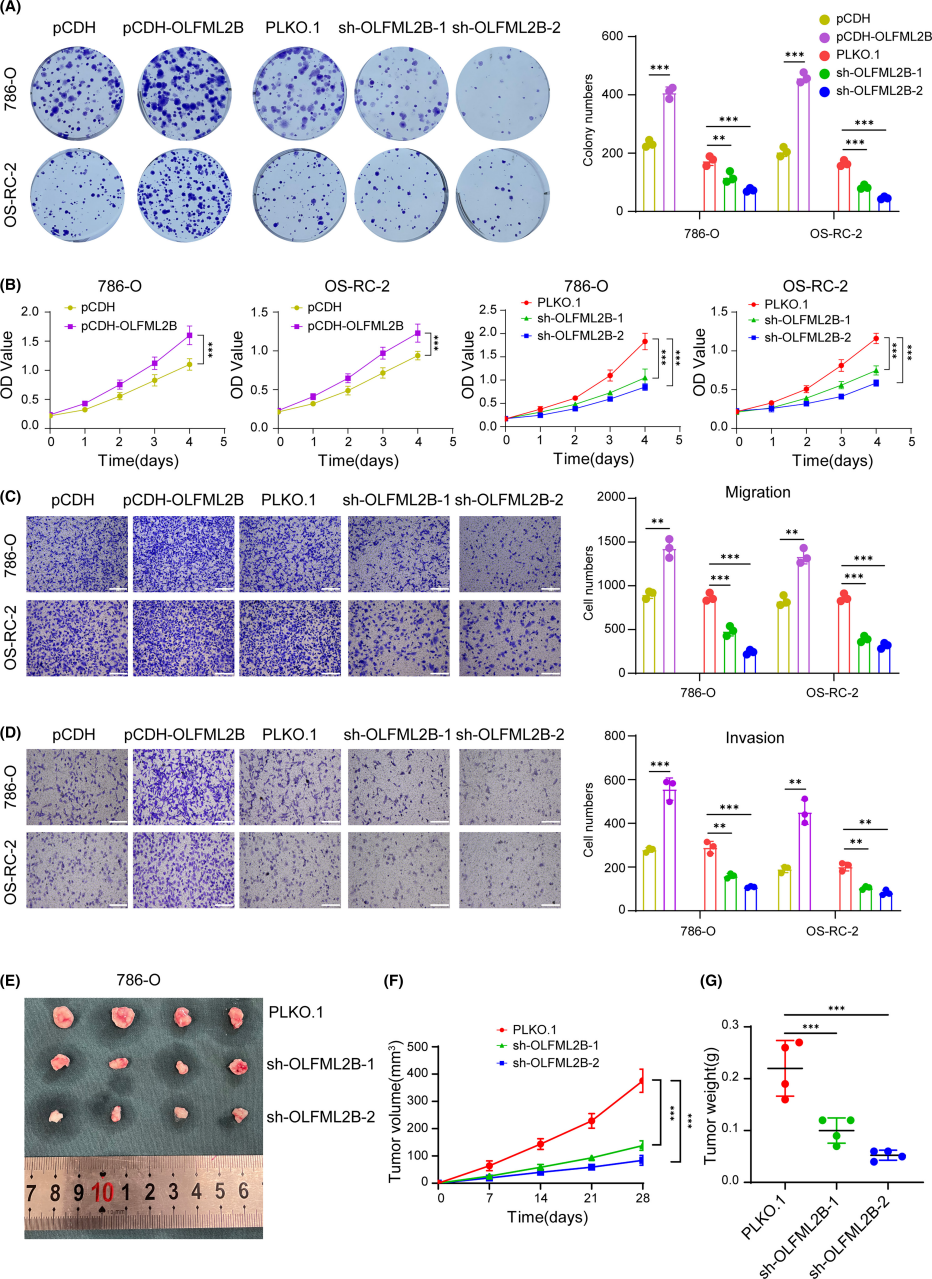
OLFML2B promoted clear cell renal cell carcinoma (ccRCC) progression in vitro and in vivo. (A) Colony formation assays were performed in 786‐O and OS‐RC‐2 cells with OLFML2B overexpression or silence. (B) CCK‐8 assays were implemented to detect 786‐O and OS‐RC‐2 cells viability. (C) Transwell migration assays were applied in ccRCC cell lines (scale bar: 25 mm). (D) Transwell invasion assays were applied in ccRCC cell lines (scale bar: 25 mm). (E–G) Stably silencing 786‐O cells and transfected by corresponding control plasmids cells were injected subcutaneously into nude mice. Isolated tumors were shown and their volume and weight were calculated. Data were shown as the mean ± SD from three repeated experiments. **p < 0.01; ***p < 0.001 were calculated using a two‐sided Student *t*‐test.

To identify the role of OLFML2B in ccRCC, we designed functional experiments in 786‐O and OS‐RC‐2 cell lines, which stably overexpressed or silenced OLFML2B as mentioned above (Figure S2). According to the colony formation assays and CCK‐8 assays' results, overexpressing OLFML2B could accelerate ccRCC cell proliferation, while knocking down OLFML2B might suppress the proliferative ability of 786‐O and OS‐RC‐2 cells (Figure 9A,B). Next, transwell migration and invasion assays were implemented to demonstrate that overexpression of OLFML2B obviously facilitated ccRCC cell migration and invasion. However, silencing OLFML2B showed the opposite effect (Figure 9C,D). Consistently, xenograft experiments in nude mice supported our previous conclusion as well. In 786‐O cells, OLFML2B knockdown greatly inhibited the growth rate of xenograft tumors (Figure 9E,F), and the tumor weight in the OLFML2B knockdown group was significantly lower compared to the control group (Figure 9G). These findings convincingly reveal the oncogenic effects of OLFML2B on ccRCC in vitro and in vivo.

## 4 DISCUSSION

As the most common type of renal cancer with a poor prognosis, ccRCC has been troubling patients and clinicians due to its recurrence and distant metastasis.^29^ The therapeutic effect of ccRCC has been significantly improved in recent decades, and immunotherapies such as PD1, PDL1, and cytotoxic T‐lymphocyte‐associated protein 4 (CTLA‐4) inhibitors have been approved for clinical treatment.^9^, ^30^, ^31^ However, there are still a large number of patients whose therapeutic effects are not obvious and who even eventually develop resistance.^10^, ^32^ Therefore, exploring new potential biomarkers is of vital importance for the treatment and management of ccRCC patients.

In this study, individuals with ccRCC from TCGA were allocated into four clusters based on prognostically associated EMT‐related genes. Subsequently, the survival probability, clinical characteristics, and mutation burden of each cluster were analyzed. Furthermore, a prognostic signature consisting of seven genes associated with EMT‐related genes was generated. Patients were categorized into high‐risk or low‐risk groups accordingly. The performance of the signature was assessed by analyzing Kaplan–Meier survival curves and ROC curves. Results demonstrated the strong predictive capabilities of the aforementioned signature, which was validated in both the ICGC and ArrayExpress databases. Additionally, we evaluated immune cell infiltration in both high‐ and low‐risk groups along with the drug resistance of prognostic genes. Finally, functional in vitro and in vivo experiments were conducted to confirm the role of the prognostic gene OLFML2B.

Numerous studies have described the important regulatory role of EMT in promoting tumor invasion and migration, and the relationship between EMT status and tumor metastasis ability has been proved. Trimboli et al.^33^, ^34^ showed that in genetically engineered mice with breast cancer, EMT events occurred in a large part of the tumors caused by the Myc gene. Xiao et al.^35^ indicated that by regulating the EMT state induced by TGF‐β1, the invasion and migration of colorectal cancer cells were significantly inhibited.^36^ Changes in EMT markers were observed in a variety of tumors, including positive expression of interstitial markers such as Vimentin, N‐cadherin, and α‐smooth muscle actin (A‐SMA),^37^, ^38^ and a lack of epithelial marker E‐cadherin. Choi et al.^39^ showed that increased mesenchymal markers ZEB1 and ZEB2 were observed in advanced invasive bladder cancer, but this change was not found in non‐muscular invasive bladder cancer.^40^ Nitta and colleagues suggested that the cadherin transmembrane protein transition from E‐cadherin to N‐cadherin was independently associated with tumor invasion and metastasis.^41^ Japanese scholar Yamada found that EMT‐induced transcription factors and mesenchymal markers Twist 1 and ZEB‐2 were significantly overexpressed in liver cancer, and patients with mesenchymal tumors were more likely to have early recurrence than those with epithelial tumors.^42^ The transcriptional inhibitors Snail and Slug promoted tumor progression by regulating EMT and antagonizing P53‐induced apoptosis, leading to drug resistance in cancer cells. Zhang et al.^43^ found that overactivation of ZEB1 promotes tumor radiation resistance in vivo and in vitro in the radiation‐resistant subsets of breast cancer, suggesting that EMT may be related to the resistance of breast cancer to radiotherapy and chemotherapy.^44^ However, few studies have explored the regulatory mechanism of EMT‐related genes in ccRCC. Based on the ccRCC samples of TCGA, we identified and constructed the prognostic signature of EMT‐related genes, verified their predictive performance, and provided new ideas for the development of potential biomarkers and targeted therapy research. Recently, a large number of studies on the prognosis of ccRCC have been reported,^45^, ^46^, ^47^, ^48^ but they have paid less attention to the molecular subtypes of ccRCC. We believe that typing or clustering can better help us understand the prognostic effect of EMT‐related genes on ccRCC. In addition, considering the regulatory effect of EMT‐related genes on drug resistance in tumor cells, we further analyzed the drug sensitivity of prognostic genes and found that these genes have sensitivity and resistance to certain drugs, which should be considered by clinicians in the future. Most importantly, almost all manuscripts emphasize bioinformatics analysis while ignoring the most important verification experiments, which is our greatest advantage. Cell function and animal experiments can make our manuscripts more credible and reliable.

In addition, it is notable that the functional relevance of COL5A1 in ccRCC has previously been documented. Furthermore, overexpression of this gene is associated with enhanced progression and metastasis of ccRCC and lower survival rates.^28^ SHROOM3 and SERPINF1 play a role in kidney transplantation and chronic kidney disease,^49^, ^50^ while RCN3 and NTM are potential prognostic molecules for non‐small cell lung cancer and ovarian cancer,^51^, ^52^ but they have not been explored in kidney tumors. The role of OLFML2B and SYLT1 in renal tumors remains unclear. Olfactomedin‐like 2B (OLFML2B) belongs to the family containing olfactory regulatory domains. One of their common features is that the C‐terminal of the protein contains an olfactory regulatory domain.^53^ Many studies have proved that *OLFML2B* is an important oncogene involved in the regulation of a variety of cancers. Liu et al. indicated that OLFML2B exhibited significant upregulation in gastric cancer and was implicated in the modulation of diverse biological events, including cell growth, cell cycle regulation, and apoptosis through the M/G1 transition pathway.^54^ Huang and colleagues suggested that *OLFML2B* and related genes predict prognosis in colorectal cancer patients by m^6^A modification.^55^ Moreover, Ren and Zhao et al. suggested that OLFML2B can predict tumor prognosis by regulating the tumor immune microenvironment and maybe a potential target for future therapy.^56^, ^57^ However, the role of OLFML2B in ccRCC has not been explored. Here, we proved that OLFML2B contributed to the proliferation and metastasis of ccRCC, affirming its prognostic efficacy for ccRCC patients through colony formation assays, EdU assays, and xenograft experiments, as well as provided new ideas for clinical work. However, the specific downstream mechanism of OLFML2B regulating ccRCC deserves further study.

## 5 CONCLUSION

In conclusion, we successfully constructed a prognostic signature of EMT‐related genes containing seven genes, which can be used as an independent prognostic factor for ccRCC. In addition, we evaluated the predictive power of the model on clinical characteristics and immune microenvironment and analyzed the drug sensitivity of prognostic genes. Finally, functional experiments verified that OLFML2B can promote tumor proliferation and metastasis and provide potential therapeutic targets for the diagnosis and treatment of ccRCC.

## AUTHOR CONTRIBUTIONS

**Yue Ge:** Visualization (lead); writing – original draft (lead). **Sheng Ma:** Software (supporting). **Junbiao Zhang:** Validation (equal); visualization (equal). **Zezhong Xiong:** Software (supporting); supervision (supporting). **Beining Li:** Supervision (supporting); validation (supporting). **Siquan Ma:** Software (equal); writing – review and editing (equal). **Bo Liu:** Writing – review and editing (supporting). **Xiangyang Yao:** Formal analysis (equal); software (equal). **Zhihua Wang:** Resources (equal); writing – review and editing (lead).

## CONFLICT OF INTEREST STATEMENT

The authors declare that the research was conducted in the absence of any commercial or financial relationships that could be construed as a potential conflict of interest.

## FUNDING INFORMATION

This study was supported by The National Natural Science Foundation of China (82173068, 81974400).

## ETHICS STATEMENT

The present study utilized 14 pairs of ccRCC tissues and adjacent normal tissues obtained from the Department of Urology, Tongji Hospital, Tongji Medical College, Huazhong University of Science and Technology (Wuhan, China). All patients provided informed consent prior to participation in this study. The research was approved by the Ethics Committee of Tongji Medical College, Huazhong University of Science and Technology.

## Supporting information


Figure S1.

Figure S2.

Table S1.

Table S2.

Table S3.

Table S4.

Data S1:

## Data Availability

The publicly available datasets analyzed in this study were available in the TCGA database (https://xenabrowser.net), ICGC (RECA‐EU/FR) (https://dcc.icgc.org/projects/RECA‐EU), and the ArrayExpress database (E‐MTAB‐1980 dataset) (https://www.ebi.ac.uk/arrayexpress/experiments/E‐MTAB‐1980/). Experimental details are available in the Supplementary methods.
