# Supplementary figures and images for: Integrating bioinformatic analysis and detailed experiments reveal an EMT‐related biomarker for clear cell renal cell carcinoma
Source: Cancer Med. 2023 Sep 7;12(18):19320–36. doi: 10.1002/cam4.6504 (PMC10557903; doi:10.1002/cam4.6504)

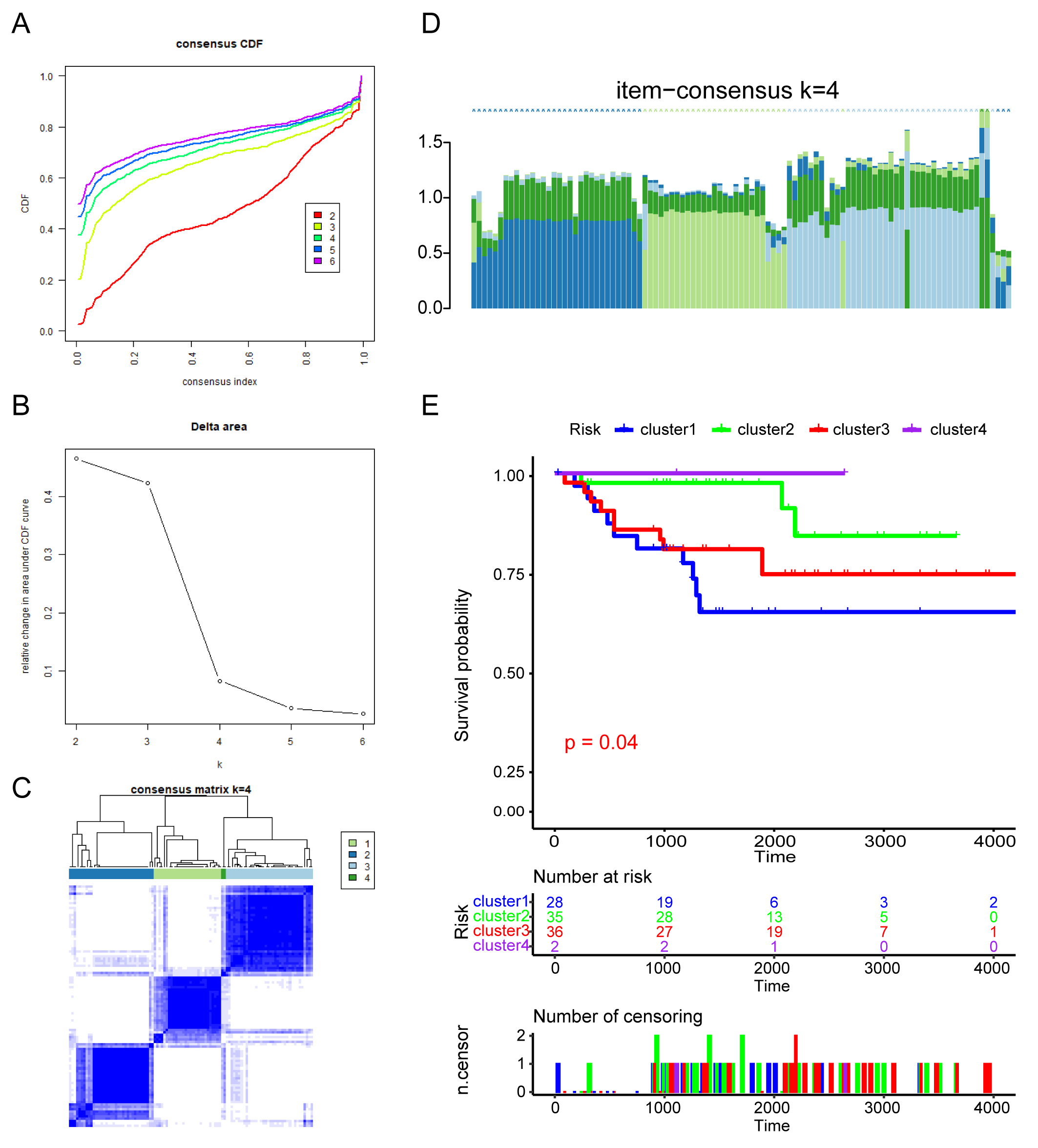

Supplement: Supplementary file 1 — Figure S1. [file CAM4-12-19320-s005.tif]

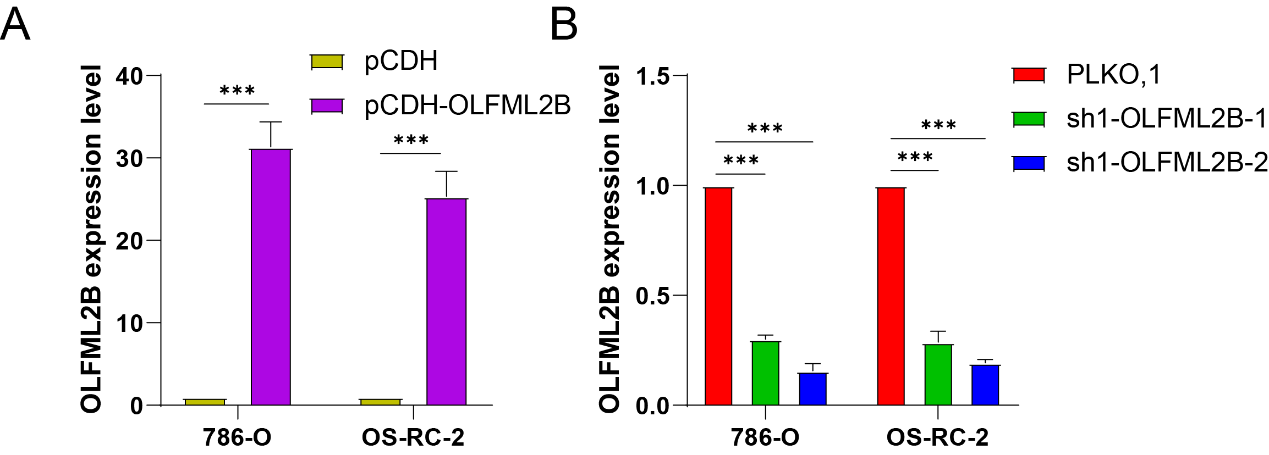

Supplement: Supplementary file 2 — Figure S2. [file CAM4-12-19320-s006.tif]
