# Supplementary material for: Integrating bioinformatic analysis and detailed experiments reveal an EMT‐related biomarker for clear cell renal cell carcinoma
Source: Cancer Med. 2023 Sep 7;12(18):19320–36. doi: 10.1002/cam4.6504 (PMC10557903; doi:10.1002/cam4.6504)
Supplement: Supplementary file 7 — Data S1: [file CAM4-12-19320-s003.docx]

**Supplementary methods**

### Clustering of ccRCC samples

The R package ConsensusClusterPlus was used for consistent clustering on the ccRCC samples from the TCGA database.  We divided the samples into seven clusters between k=2 and k=8 by using Pearson correlation coefficients and hierarchical clustering algorithms. The results were derived from 100 repeated sampling on 80% of the sample. In order to determine the optimal cluster number, a consistent cumulative distribution function (CDF) and a delta area diagram were used, both of which presented high consistency and stable areas under the CDF curve.

**Constructing and validating prognostic signature**

EMT-related genes with high significance in univariate Cox regression were subsequently used in multivariate Cox regression to build a prognostic signature. Using the following risk formula to calculate the risk score for each individual: (exp mRNA_1_ × coef mRNA_1_) + (exp mRNA_2_ × coef mRNA_2_) + ... + (exp mRNA_N_ × coef mRNA_N_). According to the "survival_cutpoint" function of the R software, the optimal cut-off value was determined, and ccRCC patients with TCGA were divided into high-risk and low-risk group. Kaplan-Meier survival and the receiver operating characteristic (ROC) analysis was performed to evaluate the power of the prognostic signature and was further validated in the external dataset ICGC and ArrayExpress. Moreover, an analysis of ccRCC survival probability was conducted using a nomogram based on prognostic signature and clinical characteristics.

**Quantitative reverse transcription PCR (qRT-PCR)**

The TRIzol reagent (Thermo Fisher Scientific, USA) was used to extract total RNA from RCC cells. In addition, reverse transcription was implemented by the Prime-Script™ RT Reagent Kit (TAKARA, China) and quantitative PCR was performed with 2×ChamQ Universal SYBR qPCR Master Mix* (Vazyme, China). OLFML2B expression was normalized to that of GAPDH. The specific primers (5’-3’) used for qPCR were as follows. (GAPDH-F: ACAACTTTGGTATCGTGGAAGG, GAPDH-R: GCCATCACGCCACAGTTTC; OLFML2B-F: AAGCCTCGGCTGCTAGTTC, OLFML2B-R: GTTGTCCGCCTCGTTTTGC)

**Colony formation assay**

In colony formation assays, approximately 1,000 786-O cells or 1,500 OS-RC-2 cells were seeded into six-well plates and incubated for 2 weeks. The numbers of cell colonies stained by crystal violet were calculated and analyzed.

**CCK-8 assay**

A total of 1,500 of 786-O cells or 2,000 of OS-RC-2 cells were seeded into 96-well plates with 100 μL of cell suspension in each well. Cell viability was assessed using CCK-8 assays (Dojindo, Japan) after 24 hours, 48 hours, 72 hours, and 96 hours of culture. The 450 nm absorbance measurement by a microplate reader (Thermo Fisher Scientific, USA) was performed after adding 10 μL CCK-8 reagent to each well and incubating for 90 minutes. Each experiment included five replicates and was repeated three times.

**Transwell assay**

The upper chambers of 24-well Transwell plates were plated with 6.0 × 104 786-O cells or 9.0 × 104 OS-RC-2 cells suspended in 200 mL FBS-free medium (Cat# 3422, Corning Costar, USA), and 600μL RPMI-1640 with 10% FBS was added to the lower chamber. For migration assay, after 12 hours incubation, cells in upper chambers were fixed by 1ml methyl alcohol and stained with crystal violet for 30 minutes. In addition, invasion assays were conducted by coating Transwell membranes with Matrigel before plating cells. After 24 hours incubation, cells in upper chambers were fixed by 1ml methyl alcohol and stained with crystal violet for 30 minutes. Finally, the ccRCC cells were captured by inverted microscope and calculated in three random fields.

**Western blot and antibodies**

The detailed protocols for western blot were explained in our previous research.[24] We used the following antibodies in this study: anti-β-actin (66009-1-lg, Proteintech), anti-N-Cadherin (66219-1-lg, Proteintech), anti-E-Cadherin (60335-1-lg, Proteintech), anti-Vimentin (60330-1-lg, Proteintech), goat anti-mouse IgG HRP (SA00001-1, Proteintech), and goat anti-rabbit IgG HRP (SA00001-2, Proteintech).
